# Gel immersion endoscopic mucosal resection for small gastric neoplastic lesions: A pilot study

**DOI:** 10.1002/deo2.70004

**Published:** 2024-08-29

**Authors:** Kosei Hashimoto, Yuji Ino, Hiroaki Ishii, Satoshi Shinozaki, Yoshimasa Miura, Edward J. Despott, Tomonori Yano, Hironori Yamamoto

**Affiliations:** ^1^ Department of Medicine Division of Gastroenterology Jichi Medical University Tochigi Japan; ^2^ Shinozaki Medical Clinic Tochigi Japan; ^3^ Royal Free Unit for Endoscopy The Royal Free Hospital and UCL Institute for Liver and Digestive Health London UK

**Keywords:** endoscopic hemostasis, gastroscopy, gel immersion endoscopy, stomach neoplasms, underwater endoscopic mucosal resection

## Abstract

Gastric endoscopic mucosal resection is challenging due to the slippery mucosa, abundant blood vessels, and the presence of mucus. We developed gel immersion endoscopy to secure the visual field, even in a blood‐filled gastrointestinal lumen in 2016. Clear gel with appropriate viscosity, instead of water, can prevent rapid mixture with blood and facilitate identification of the culprit vessel. We further optimized the gel for endoscopic treatment, and the resultant product, Viscoclear (Otsuka Pharmaceutical Factory) was first released in Japan in 2020. The viscosity of this gel has been optimized to maximize endoscopic visibility without compromising the ease of its irrigation. The aim of this study is to clarify the effectiveness of gel immersion endoscopic mucosal resection for small‐sized early gastric neoplasms. Seven lesions in seven patients were treated by gel immersion endoscopic mucosal resection. The size of all lesions was under 10 mm. The median procedure time was 4.5 min. Intraoperative bleeding occurred in four of seven lesions immediately after snare resection and was easily controlled by endoscopic hemostatic forceps during the gel immersion endoscopy. The R0 resection rate was 100%. In conclusion, gel immersion endoscopic mucosal resection may be a straightforward, rapid, and safe technique for resecting superficial gastric neoplasms <10 mm in diameter.

## INTRODUCTION

**FIGURE 1:**
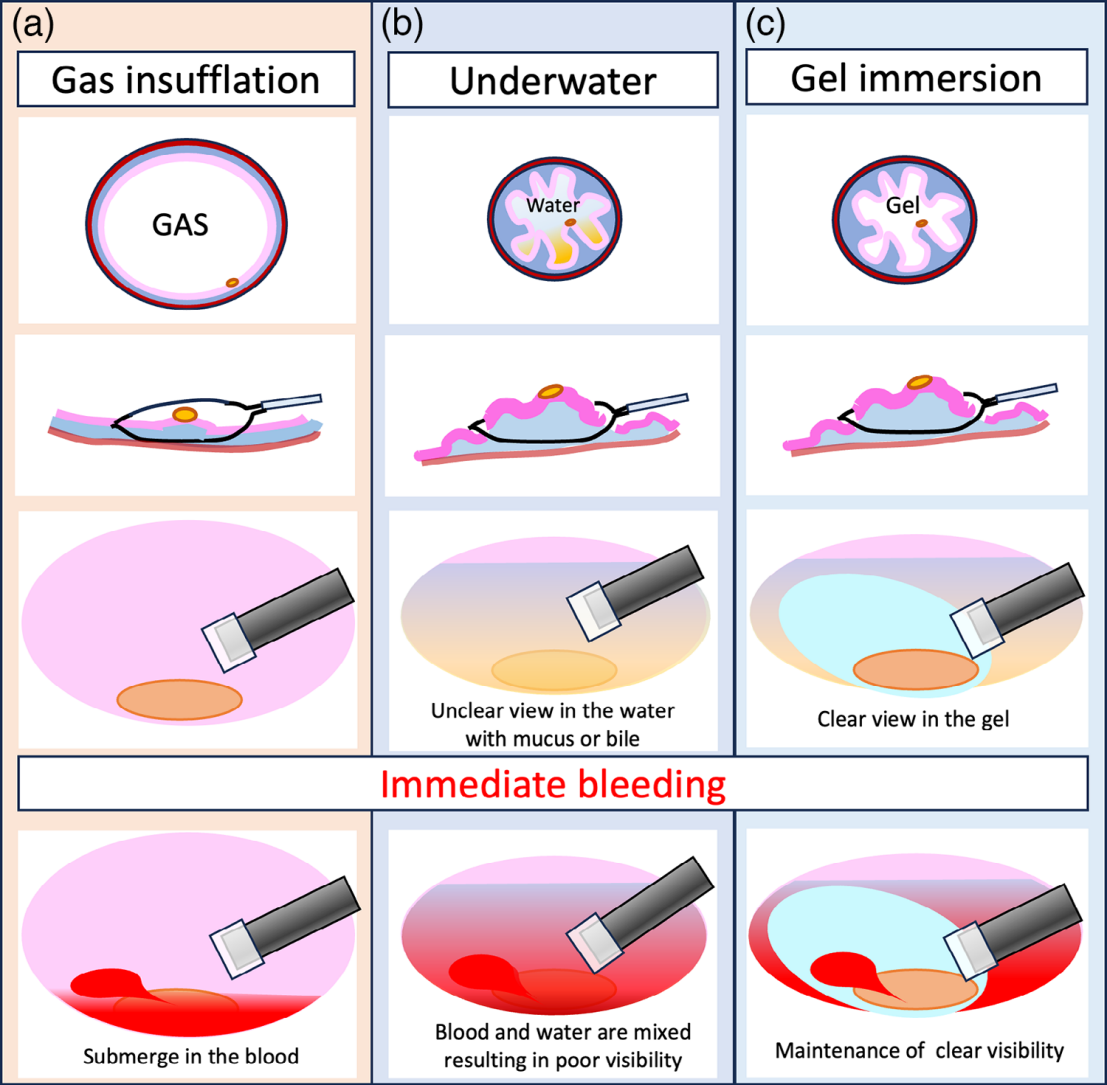
Procedure of gel immersion endoscopic mucosal resection: (a) In conventional endoscopic mucosal resection, the snaring is difficult caused of the slippery mucosa of the air‐insufflated and distended stomach. At intraprocedural bleeding, the bleeding point is difficult to determine in a submerged area; (b) In underwater endoscopic mucosal resection, the snaring is facilitated by a shrunken gastric lumen. However, intraprocedural bleeding in submerged areas causes a rapid mixture of water and blood; (c) The gel immersion endoscopic mucosal resection facilitates snaring under a clear visualization even in the presence of gastric mucus in the shrunken gastric lumen. It also facilitates endoscopic hemostasis for intraprocedural bleeding just after the resection by visualizing a bleeding point because injected gel hardly mixes with blood.

The evolution of endoscopic technology such as image‐enhanced endoscopy and chromoendoscopy facilitates the detection of even small gastric neoplasms. The Japanese Guidelines for endoscopic submucosal dissection (ESD) and endoscopic mucosal resection (EMR) for early gastric cancer recommends ESD/EMR as an absolute indication for differentiated mucosal gastric cancer ≦2 cm without findings of ulceration.^1^ Conventional EMR sometimes cannot accomplish en bloc resection for superficial gastric neoplasms. Unlike the EMR for colonic neoplasms, gastric neoplasms are difficult to snare due to the slippery gastric mucosa, and therefore the risk of inadvertent piecemeal resection is comparatively high (Figure 1a). Despite the reported effectiveness of underwater EMR for colonic neoplasms, maintaining clear visualization during underwater conditions can be challenging.^2^, ^3^ Notably, the stomach has a wider lumen and is more vascular than the colon; furthermore, gastric mucus and any retained gastric residue may cloud the underwater view (Figure 1b). Therefore, underwater EMR within the stomach is much more challenging than it is within the colon. Intraprocedural active bleeding just after gastric EMR impedes visibility and makes hemostasis difficult to achieve, especially within the wide lumen, coupled with the presence of mucus. For these reasons, gastric superficial neoplasms have been resected by ESD rather than EMR, even if the tumor is otherwise small enough to be treated with EMR. ESD could be technically challenging and time‐consuming and requires the use of dedicated devices including knives and traction devices that are more expensive than the snares used in EMR.

We developed a gel immersion endoscopy to secure the visual field in the blood‐filled gastrointestinal lumen in 2016.^4^ At emergency endoscopy for gastrointestinal bleeding, blood, clots and residue hinder the visual field and may prevent endoscopists from identifying the bleeding point. Although the water immersion method may be attempted to try to secure a better view, the rapid mixing of blood with water still leads to clouding, and any adequate views obtained are usually only transient. The substitution of water with gel of adequate viscosity can improve the visual field by creating a viscous, clear barrier that prevents rapid mixing with blood, thus facilitating the identification of the bleeding point. We further optimized the gel for endoscopic treatment,^5^ and the resultant product, Viscoclear (Otsuka Pharmaceutical Factory) was first released for use in Japan in 2020. The viscosity of this gel has been optimized to maximize endoscopic visibility without compromising the ease of its irrigation. Additionally, it has low electrical conductivity (due to the minimal presence of electrolytes), allowing for efficient use of high‐frequency electrocautery forceps with settings similar to those used with gaseous luminal insufflation.^5^ There are few reports regarding the gel immersion endoscopic mucosal resection (GIEMR) for gastric superficial neoplasms. The aim of this study is to clarify the effectiveness of GIEMR for small‐sized early gastric neoplasms.

## PROCEDURE

### Study population

This was a retrospective observational study involving patients with a superficial gastric neoplasm ≦10 mm treated by GIEMR who were diagnosed by preoperative biopsy regardless of the shape. The indication of GIEMR was as follows: (1) 10 mm or less in size; (2) no visible stigmata of deep invasion; (3) endoscopic snaring can be done with adequate margins. All endoscopic resections were recorded on video. From December 2022 to March 2023, GIEMR was performed for seven gastric neoplasms at Jichi Medical University Hospital. Their medical records and videos were retrospectively reviewed. The Institutional Review Board approved this retrospective review (ID#19‐112).

### Procedure for GIEMR

**FIGURE 2:**
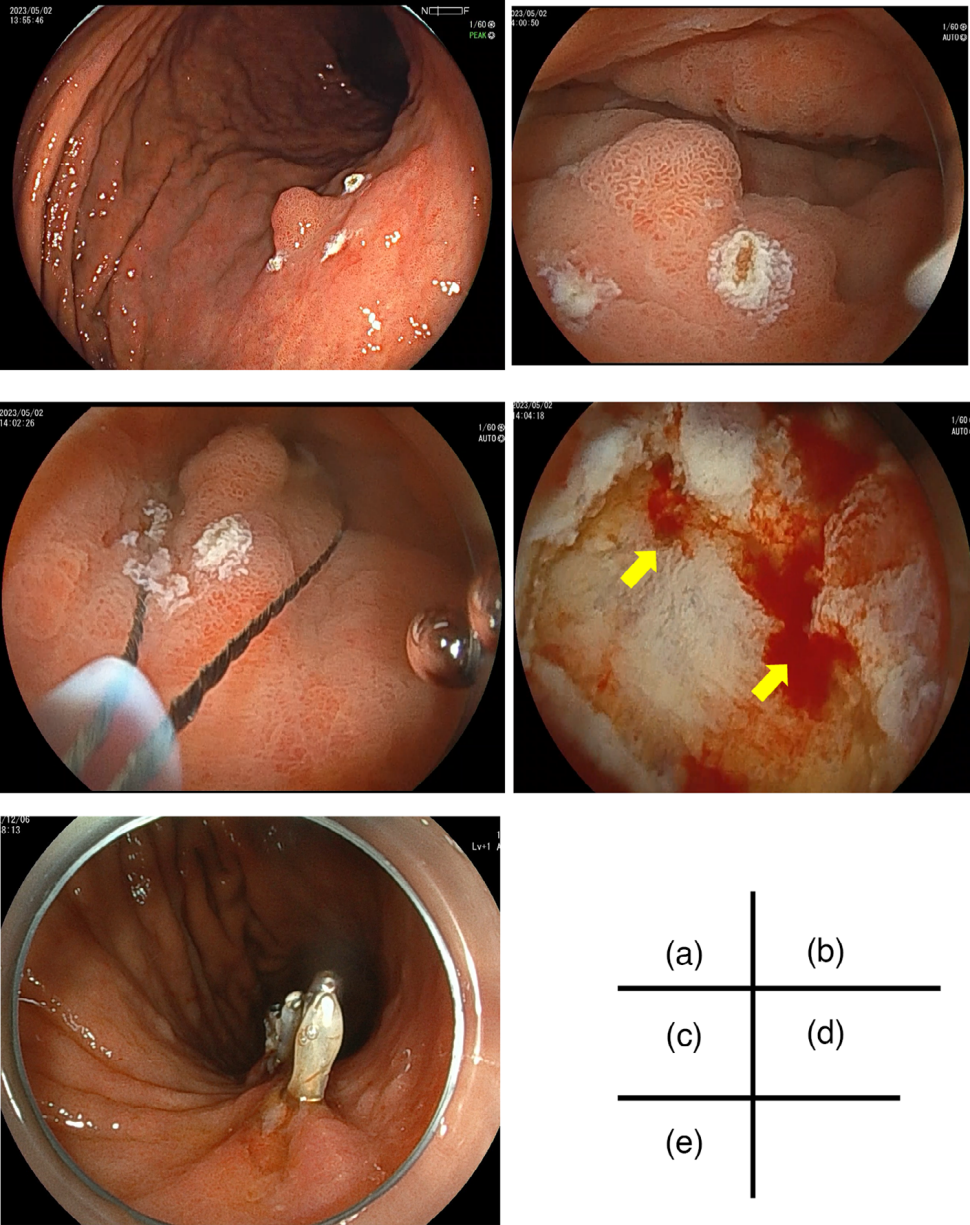
Endoscopic images of gel immersion endoscopic mucosal resection: (a) Marking with electrocauterization using snare tip; (b) Minimum Viscoclear injection to recognize the lesion and markings after complete sucking of luminal air; (c) snaring and resection by involving surrounding markings; (d) Bleeding points (yellow arrows) are recognized easily without mixing blood that enables endoscopic electrocauterization; (e) closure of mucosal defect that is facilitated by gel immersion condition where mucosal defect does not enlarge unlike air insufflated condition.

First, snare‐tip electrocautery markings are placed around the target lesion, to ensure accurate delineation. After aspiration of luminal gas from the stomach, a gel is gently irrigated into the gastric lumen. A low intraluminal pressure allows involution of gastric folds and relative thickening of the underlying submucosal layer, while the muscularis propria remains linear. This phenomenon omits the need for submucosal injection. After confirming the snare‐capture of whole target lesions (including initial markings), en bloc resection is performed with electrocautery. The mucosal defect is then carefully inspected under gel immersion conditions. Any visible blood vessels and/or active bleeding are immediately treated with further diathermy. The viscosity of the gel facilitates visualization of any bleeding points and additional slow‐irrigation of gel can be performed as needed (Figure 1c). Finally, the resultant mucosal defect is closed using endoclips while maintaining gel immersion (Figure 2, Video S1). The amount of Viscoclear used was 100–400 mL per procedure.

### Endoscopes and devices

A magnifying/therapeutic endoscope (EG‐L600ZW7, EG‐L580RD, or EG‐840T; Fujifilm), a carbon dioxide insufflator (GW‐1 or GW‐100; Fujifilm), a transparent distal attachment (D‐201‐11804; Olympus) and a diathermy unit (Vio3 or Vio300; ERBE) were used. Our proprietary gel (Viscoclear) was used instead of water. For resection, a 15‐mm Rota snare (Medi‐Globe GmbH) or a 20‐mm Spiral snare (Olympus) was used. Reopenable endoclips (SureClip; Micro‐Tech Co. Ltd.) and standard clips (EZ clip; Olympus) were used to close the mucosal defect.

### Evaluation of GIEMR

Resection time (A) was defined as the time between the start of gel irrigation and the completion of snare resection. Hemostasis time (B) was defined as the time between the completion of snare resection and the achievement of hemostasis. Procedure time (A+B) was defined as the time between the start of gel irrigation and the achievement of hemostasis. Closure time was defined as the interval between the completion of snare resection or achievement of hemostasis and closure of the mucosal defect by endoclips. En‐bloc resection was defined as single‐piece lesion resection. R0 resection was defined as an en‐bloc resection with negative pathological margins. Intraprocedural bleeding was defined as persistent bleeding from the mucosal defect immediately after the resection. Delayed bleeding was defined as a decrease of hemoglobin level ≧2 g/dL or any bleeding requiring transfusion or endoscopic hemostasis within 14 days after the GIEMR. Intraprocedural perforation was defined as visualization of the peritoneal cavity through damaged muscularis propria during GIEMR, and delayed perforation was defined as the presence of free air on a computed tomography scan with peritonism after GIEMR.

## RESULTS

The characteristics of seven lesions in seven patients who underwent the GIEMR are shown in Tables 1 and 2. The size of all lesions was under 10 mm. The median procedure time was 4.5 min. Intraprocedural bleeding occurred in four of seven cases, just after the snare resection; hemostasis with coagulation forceps was easily achieved within 4 min in gel immersion conditions. At pathological analysis, one specimen did not show any neoplastic lesion; this was probably caused by the removal of the very small lesion by preoperative biopsies. Except for the lesion, the pathological diagnoses were adenoma or adenocarcinoma, and the stage of adenocarcinoma was T1a. R0 resection was achieved in all lesions. The amount of Viscoclear used in one session was approximately 100–400 mL.

**TABLE 1:** Baseline characteristics of patients and lesions.

Number of lesions, *n*	7
Number of patients, *n*	7
Age, years, median	75 (68–85)
Gender, male: female, *n*	4:3
Tumor location, *n*	
Lesser curvature	4
Greater curvature	2
Remnant stomach	1
Macroscopic type, *n*	
0‐IIa	6
0‐IIc	1
Tumor diameter, mm, median (range)	5 (5–9)
Resected specimen diameter, mm, median (range)	20 (14–25)
Procedure time (A+B), min, median, (range)	4.5 (2.6–8.3)
Resection time (A), min, median (range)	3.8 (2.1–5.5)
Perforation, *n* (%)	0 (0%)
Intraprocedural bleeding, *n* (%)	4 (57%)
Hemostatic time for bleeding (B) (*n* = 4), min, median (range)	2.6 (1.0–3.4)
Delayed bleeding, *n* (%)	0 (0%)
Pathological findings, *n* (%)	
Adenocarcinoma	4
Adenoma	2
Non‐neoplasm	1
En‐bloc resection, *n* (%)	7 (100%)
R0 resection, *n* (%)	7 (100%)

**TABLE 2:** Characteristics of lesions resected.

Patient	Macroscopic type	Tumor location	Tumor diameter (mm)	Diameter of resected specimen (mm)	Resection time (A) (min)	Hemostatic time for intraprocedural bleeding (B) (min)	Procedure time (A)+(B) (min)	Closure time (min)	Histopathology
1	0‐IIa	M/LC	5	22 × 19	5.5	2.8	8.3	2.7	No neoplasm
2	0‐IIa	L/GC	5	20 × 20	3.8	3.4	7.3	7.7	tub1 (T1a)
3	0‐IIc	U/LC	5	25 × 11	2.1	2.4	4.5	10.5	tub1 (T1a)
4	0‐IIa	Remnant stomach	6	14 × 11	4.6	No bleeding	4.6	10.4	Adenoma
5	0‐IIa	L/GC	9	18 × 16	3.5	1.0	4.5	7.5	tub1 (T1a)
6	0‐IIa	M/LC	7	17 × 15	3.8	No bleeding	3.8	5.8	tub1 (T1a)
7	0‐IIa	L/LC	5	24 × 22	2.6	No bleeding	2.6	5.4	Adenoma

U:upper part, M: middle part, L: lower part, GC: greater curvature, LC: lesser curvature

(A) Resection time: time between the start of gel irrigation and completion of snare resection

(B) Hemostatic time: time between completion of snare resection and achievement of hemostasis

(A) + (B) Procedure time: time between the start of gel irrigation and achievement of hemostasis

## DISCUSSION

This pilot study suggested that GIEMR facilitates en‐bloc resection with efficient intraprocedural endoscopic hemostasis and a short overall procedural time. Before the development and dissemination of gastric ESD, EMR for gastric neoplastic lesion (<10 mm) was performed with a poor en‐bloc resection rate (62%–87%) even if various devices, including EMR with cap (EMR‐C) and EMR with ligation device (EMR‐L), were used. In conventional EMR, piecemealed resection or R1 resection can be caused by slippage of the snare at strangulation and by poor recognition of the distal tip in an inflated stomach. The difficulty of en‐bloc resection is also influenced by tumor location such as the lesser curvature or posterior wall, where the approach to the lesion tends to be tangential/vertical. Although aspiration methods, including EMR‐C and EMR‐L, enable a vertical approach, the size of the aspiration area is limited, and inadvertent inclusion of the muscularis propria may lead to perforation.

The remarkable progress of image‐enhanced endoscopy has allowed earlier detection of gastric neoplasia, allowing endotherapy of such lesions at even smaller sizes. It is well known that gastric ESD in the upper part of the stomach requires sophisticated skills, expensive devices, and long procedure times. However, the mainstay of endoscopic therapy for superficial gastric neoplasm has been ESD, even if the lesion size was sufficiently small for EMR. To overcome these limitations of gastric ESD, we introduced GIEMR to resect small gastric neoplastic lesions. GIEMR with our gel enables clear visualization to facilitate snaring with low gel volumes. The omission of gaseous insufflation during GIEMR enables gastric mucosal convolution, thus facilitating the safe resection of lesions that are larger than the snare diameter. For gastric ESD, a vertical approach to lesions located on the greater curvature, where water tends to accumulate is particularly difficult. However, on the contrary, the vertical approach is beneficial for performing the GIEMR as long as the lesion is submerged in the gel. If luminal gas is adequately aspirated, GIEMR can also be feasible to treat small lesions located on the lesser curvature. Furthermore, GIEMR is useful to resect lesions located near the pyloric ring, because unlike water immersion, which is difficult to achieve in this location, the gel's viscosity facilitates the retainment of gel immersion conditions.^6^ A recent Japanese study including 37 gastric neoplasms in patients with familial adenomatous polyposis compared underwater EMR with conventional EMR.^7^ The procedure time of the UEMR group was significantly shorter than that of the conventional EMR group (*p* = 0.013). Since like underwater EMR, GIEMR does not require submucosal injection, we expect that the procedure time of GIEMR would also be shorter than conventional EMR.

The GIEMR also facilitates endoscopic hemostasis during active bleeding immediately after the snare resection. As we previously reported, gel immersion endoscopy is effective in securing the visual field, even in a blood‐filled gastrointestinal lumen. Gastric EMR has a higher risk of large intraprocedural bleeding just after the snaring than ESD, due to abundant thick blood vessels in the submucosal layer, since the EMR procedure does not include pre‐coagulation of these vessels. In the present study, 57% of patients had acute bleeding after GIEMR; these bleeding points were easily identified in the gel immersion condition. Since this gel has low electrical conductivity, if acute bleeding immediately after snaring, the bleeding point can be easily identified and cauterized by coagulation forceps in gel immersion, without switch to gaseous insufflation. Also, unlike gas‐insufflation conditions, the mucosal defect is not stretched by distension during gel immersion conditions; this facilitates clip‐closure of the narrower defect. Effective clip‐closure of highly vascularized gastric resection sites facilitated by gel immersion mitigates the risk of any delayed bleeding.

We recognized and acknowledged limitations. This is a single‐center retrospective study with a small number of patients without a control group. Although we believe the hemostasis is easier during GIEMR compared to air‐insufflated conditions, no formal comparison was performed. The selection bias based on the clinical indications may influence these results. This study included only one depressed‐type gastric tumor. Despite these limitations, we believe that gastric GIEMR facilitates endoscopic resection regardless of the location, especially in the cardia or fornix. Future comparison studies are necessary to clarify the time‐saving effectiveness and R0 resection rate compared to conventional EMR or ESD.

In conclusion, GIEMR may be a straightforward, rapid, and safe technique for resecting superficial gastric neoplasms ≦10 mm in diameter. GIEMR may be a good indication for these lesions located in the upper gastric body where conventional gastric ESD is difficult due to an inevitable vertical approach. We encourage further large studies to confirm our preliminary results.

## CONFLICT OF INTEREST STATEMENT

Tomonori Yano holds patents and is the inventor of the dedicated gel for the gel immersion method and has received royalties and honoraria from Otsuka Pharmaceutical Factory. He has received research funding and honoraria from Fujifilm and honoraria from Olympus. Hironori Yamamoto has consultant relationships with Fujifilm and received honoraria, grants, and royalties from the company. Edward J. Despott has received academic grants and speaker honoraria from Fujifilm and Olympus. The other authors declare no conflict of interest.

## ETHICS STATEMENT

The Institutional Review Board approved this retrospective review (ID#19‐112).

## Supporting information


Video S1 Procedure of GIEMR
